# Label-free imaging of trabecular meshwork cells using Coherent Anti-Stokes Raman Scattering (CARS) microscopy

**Published:** 2011-10-08

**Authors:** Tim C. Lei, David A. Ammar, Omid Masihzadeh, Emily A. Gibson, Malik Y. Kahook

**Affiliations:** 1 Department of Electrical Engineering, University of Colorado Denver, Denver, CO; 2 Department of Ophthalmology, University of Colorado Denver, Aurora, CO; 3 Department of Bioengineering, University of Colorado Denver, Aurora, CO

## Abstract

**Purpose:**

To image the human trabecular meshwork (TM) using a non-invasive, non-destructive technique without the application of exogenous label.

**Methods:**

Flat-mounted TM samples from a human cadaver eye were imaged using two nonlinear optical techniques: coherent anti-Stokes Raman scattering (CARS) and two-photon autofluorescence (TPAF). In TPAF, two optical photons are simultaneously absorbed and excite molecules in the sample that then emit a higher energy photon.  The signal is predominately from collagen and elastin. The CARS technique uses two laser frequencies to specifically excite carbon-hydrogen bonds, allowing the visualization of lipid-rich cell membranes. Multiple images were taken along an axis perpendicular to the surface of the TM for subsequent analysis.

**Results:**

Analysis of multiple TPAF images of the TM reveals the characteristic overlapping bundles of collagen of various sizes. Simultaneous CARS imaging revealed elliptical structures of ~7×10 µm in diameter populating the meshwork which were consistent with TM cells. Irregularly shaped objects of ~4 µm diameter appeared in both the TPAF and CARS channels, and are consistent with melanin granules.

**Conclusions:**

CARS techniques were successful in imaging live TM cells in freshly isolated human TM samples. Similar images have been obtained with standard histological techniques, however the method described here has the advantage of being performed on unprocessed, unfixed tissue free from the potential distortions of the fine tissue morphology that can occur due to infusion of fixatives and treatment with alcohols. CARS imaging of the TM represents a new avenue for exploring details of aqueous outflow and TM cell physiology.

## Introduction

In the conventional outflow system of the eye, aqueous humor exits the anterior chamber through the trabecular meshwork (TM) before passing into Schlemm’s canal and more distal structures. The TM region is characterized by overlapping collagen bundles that create a porous tissue populated by TM endothelial cells. These cells have been implicated in maintaining the health of the aqueous outflow system, since the number of live TM cells within the meshwork was found to be statistically lower in patients with primary open-angle glaucoma [1]. Our previous publications have demonstrated that by using two-photon autofluorescence (TPAF) and second harmonic generation (SHG) nonlinear optical microscopy, the collagen beams of the drainage system can be readily imaged without the need for exogenous fluorophores [2-6]. However, neither TPAF nor SHG can efficiently image the TM cells that reside on the collagen beams. In this study, we report that TM cells can readily be imaged without exogenous labeling at the corneal rim of a human cadaver eye using coherent anti-Stokes Raman scattering (CARS) microscopy [7-9]. We believe this is the first report of its kind and represents a new avenue for exploring details of aqueous outflow and TM cell physiology.

## Methods

### Sample preparation

A human globe from a pseudophakic 86 year old donor with no history of glaucoma was obtained from the San Diego Eye Bank (SDEB, San Diego, CA). Approval was obtained from the Colorado Multiple Institutional Review Board for the use of human material and the tenets of the Declaration of Helsinki were followed. Informed consent was obtained from donor or relatives for use in research by the SDEB. The intact globe was cut circumferentially approximately 3 mm from the corneal limbus. This anterior region was cut into quadrants, and the overlying ciliary body and iris was cut away from the TM region using spring scissors. This quadrant of corneal rim tissue was placed in a glass-bottom 35 mm dish (MatTek Corporation, Ashland, MA) with the interior surface facing down. A small glass weight placed on top of the corneal rim to maintain contact of the tissue with the glass coverslip.

### The CARS/TPAF multiphoton microscopy platform

**Figure 1:**
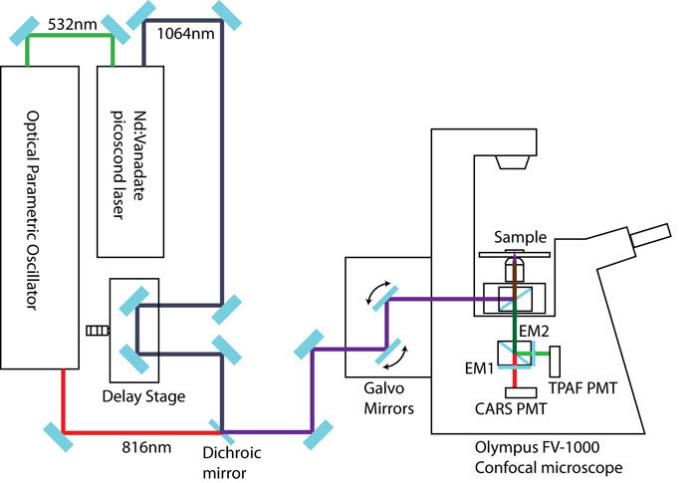
Schematic diagram of the Coherent Anti-Stokes Raman Scattering (CARS)/Two-Photon Autofluorescence (TPAF) microscope. The system consists of a Nd:Vanadate picosceond laser and an optical parametric oscillator. The scanning microscope is based on the Olympus FV-1000 confocal microscope system with two non-descanned external detectors in the epi-direction. EM1 is an emission filter to detect the CARS signal at 662 nm by the CARS PMT detector and EM2 is an emission filter to allow TPAF signal from 420 to 520 nm to be detected by the TPAF photomultiplier tube (PMT).

The CARS/TPAF images of the TM cells were acquired with a custom-built multiphoton microscopy platform optimized for CARS and TPAF imaging as shown in Figure 1. The system consists of a diode-pumped Nd:Vanadate (Nd:YVO_4_) picoseconds (ps) laser (picoTRAIN, HighQ Laser, Austria) which is capable of generating 10 Watt at 1064 nm of ~7.5ps optical pulses at a repetition rate of 80 MHz. Inside the laser, 9 Watt of the generated 1064 nm laser beam is redirected to a frequency doubling crystal to produce 4 Watt of 532 nm light with ~6 ps optical pulsewidth. The 4 Watt 532 nm laser beam is subsequently sent into an optical parametric oscillator (Levante Emerald, APE Angewandte Physik & Elektronik GmbH, Berlin, Germany) to convert the 532nm laser beam into a 1 Watt, ~6ps, 816 nm laser beam through the nonlinear optical process of difference frequency generation. The remaining 1W 1064 nm beam (Stokes) from the Nd:Vanadate laser is then optically recombined with the 816nm optical beam (Pump and Probe) and the combined laser beam is sent into an Olympus FV-1000 confocal microscope platform (Olympus, Center Valley, PA) for CARS and TPAF imaging. The optical power at the objective is 40 mW for the 816 nm laser beam and 20 mW for the 1064 nm laser beam.

The Olympus FV-1000 microscope is an inverted microscope and is equipped with four external photomultiplier detectors (PMTs) – two detectors in the epi-direction (reverse orientation) and the other two detectors in the forward directions. In this experiment, both the TPAF and CARS signals are measured in the epi-direction by collecting back-scattered photons through the objective. A dichroic mirror was used to separate the TPAF signal from the CARS signal and detected by the two epi-detectors respectively. Using an emission filter (hp470/100m-2p, Chroma Technology Corp, Bellows Falls, VT) in front of the first epi-detector, autofluorescence signal between 420 to 520 nm is detected. The CARS signal is measured with the second epi-detector with a 40 nm-wide emission filter centered at 660 nm (hq660/40m-2p; Chroma Technology). The objective used in this experiment is a 60× 1.2NA water objective (UPLSAPO 60× IR W; Olympus, Center Valley, PA) optimized for CARS and TPAF imaging. The pixel dwell time is 10 µs and the image pixel resolution is 1600×1600 for all the acquired images. A Kalman average filter of 5 times is used during image acquisitions to improve the signal-to-noise ratio of the acquired images.

### Two-Photon Autofluorescence (TPAF) microscopy theory

**Figure 2:**
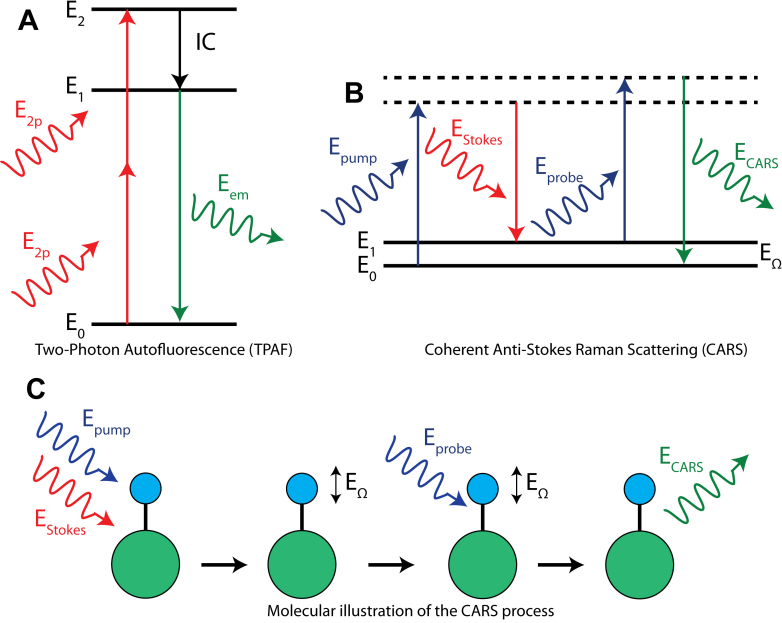
Energy diagrams of the Two-Photon Autofluorescence (TPAF) and Coherent Anti-Stokes Raman Scattering (CARS). **A**: Energy diagram of TPAF, in which an autofluorescent molecule simultaneously absorbs two optical infrared photons (*E*_2_*_p_*). After internal-crossing (IC), in which some energy is lost, the fluorescent molecule will emit a fluorescence photon (*E_em_*). **B**: Energy diagram of CARS, in which two optical photons with the photon energy difference (*E_pump_* - *E_Stokes_*) equaling to the vibrational energy of a molecule (*E*_Ω_) is used to excite the vibrational motion of the molecule. A third photon (*E_probe_*) is subsequently used to interact with the vibtational motion of the molecule, resulting in the emision of an energy-upshifted photon (*E_CARS_*). **C**: A schematic diagram illustrating the CARS process. The pump and the Stokes photons are simultaneously exciting the lipid molecule, with the energy difference between the two photons equal to the vibrational energy of the molecule bond (*E*_Ω_). Subsequent interaction of the probe photon coherently interacts with the vibrational motion of the molecule to generate a release of the CARS photon.

In human tissue, there are several endogenous molecules (collagen, elastin, and nicotinamide adenine dinucleotide [NADH]) that autofluoresce when excited by photons with the optical energy that matches the absorption spectra. Two-photon autofluorescence microscopy (TPAF) uses a femtosecond or picosecond pulsed laser in the infrared wavelength to excite the endogenous autofluorescent molecules through simultaneous absorption of two infrared photons to have the total photon energy matching the absorption energy of the endogenous molecules [2-5,10-12]. The benefits of using two infrared photons to excite the autofluorescent molecules instead of a single higher energy photon include reduced phototoxicity and much deeper penetration in biologic tissues [13]. As shown in Figure 2A, two optical photons are simultaneously absorbed by an autofluorescent molecule and the autofluorescent molecule subsequently emits an optical photon with reduced photon energy than the combined excitation energy due to energy loss through the internal conversion (IC) process. Since the TM region of the eye is mainly composed of collagen molecules in the extracellular network, capturing the fluorescent photons with the proper optical filters matching the emission spectrum of collagen in front of the photo-detector allows us to image the collagen extracellular matrix around the TM region intrinsically without the need for exogenous labeling.

### Coherent Anti-Stokes Raman Scattering (CARS) microscopy theory

CARS is another nonlinear optical microscopy technique that allows label-free imaging of lipids and other bio-molecules [7-9,14,15]. Despite the fact that CARS microscopy also uses infrared, pulsed lasers and multiple optical photons interact with the sample in the CARS process, the underlying principle of CARS microscopy is vastly different from TPAF [14,16]. CARS microscopy uses the principle of Raman scattering to measure the vibrational spectrum of the bio-molecules. The vibrational spectrum (E_Ω_ in Figure 2B,C) is the specific frequencies at which the atoms inside a molecule vibrate. These vibrational frequencies are different for different molecules and therefore the vibrational spectrum can be used to identify the molecular bond being measured. In traditional Raman spectroscopy, the optical photon impinging on a molecule can lose a fraction of the photon energy through exciting one of the vibrational motions (modes) of the molecule. Since the photon energy lost equals the vibrational energy of the molecule, the vibrational spectrum of the molecule can be formed through measuring the photon energy lost during the process. However, Raman spectroscopy is a very slow process and typically requires tens of seconds of data acquisition time which renders this technique impractical for biological imaging [14].

To speed up the imaging process, the optical nonlinear response of the material is used. As shown in Figure 2B,C, two laser beams (the pump and the Stokes beams) are used to excite the molecule by matching the photon energy difference between the two laser beams to the vibrational energy of a specific molecular bond (E_Ω_ in Figure 2B,C). This is typically the CH_2_ stretching vibrational mode at ~2850 cm^−1^ for lipid molecules [7,15]. Since the molecule is excited coherently with two synchronized laser pulses, the excitation efficiency of the vibrational mode (the CH_2_ stretch of the lipid molecules) is enhanced which significantly reduces the pixel acquisition time down to several µs. A third optical photon subsequently interacts coherently with the excited molecule to extract the stored vibrational energy to emit a CARS photon (E_CARS_, Figure 2B,C) with higher photon energy for detection.

## Results

**Figure 3:**
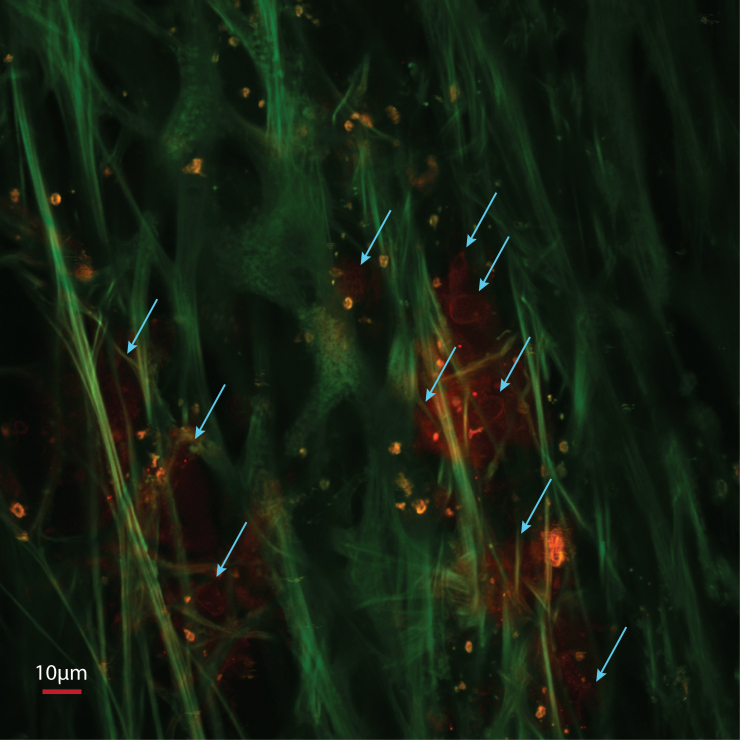
Label free imaging of the trabecular meshwork of a human cadaver eye using two photon autofluorescence (TPAF) and Coherent Anti-Stokes Raman Scattering (CARS) microscopy. (Green: TPAF, Red: CARS) The arrows indicate the TM cells that reside in the interstitial areas within the collagen extracellular matrix (EM) structure.

CARS/TPAF images are taken along the TM region in the cadaver coronal rim sample. Figure 3 shows a typical image, displaying the TPAF imaging channel in green and the CARS imaging channel in red. Due to autofluorescence of the collagen molecules, the collagen extracellular matrix shows clearly in the TPAF channel. In these images of the TM, the collagen fibers appear as smooth fiber bundles of various diameters, ranging from 1 and 10 µm. The fibers are straight with a consistent diameter, although the occasional bifurcation is visible. Qualitatively, the fiber structures are similar to those characterized previously using a commercially available two-photon microscope with a tunable femtosecond laser source [3]. In addition, the cell membrane of the TM cells is detected in the CARS channel. These cells are shown residing in the interstitial region between the collagen fiber structure (Figure 3, arrows). The size of the TM cells shown in the image are elliptical shape, with the long axis measured to be 10.4±1.2 µm and the short axis measured to be 6.9±1.1 µm (n=12). We calculate a cell density 281±59 cells/mm^2^ TM cells (n=5) in the surface region of the filtering TM. These cells have a uniformly smooth and rounded appearance, suggesting that they are healthy. In contrast, the highly autofluorescent pigment granules have a much more irregular shaped and have a considerably smaller diameter at 3.7±0.7 µm (n=12).

**Figure 4:**
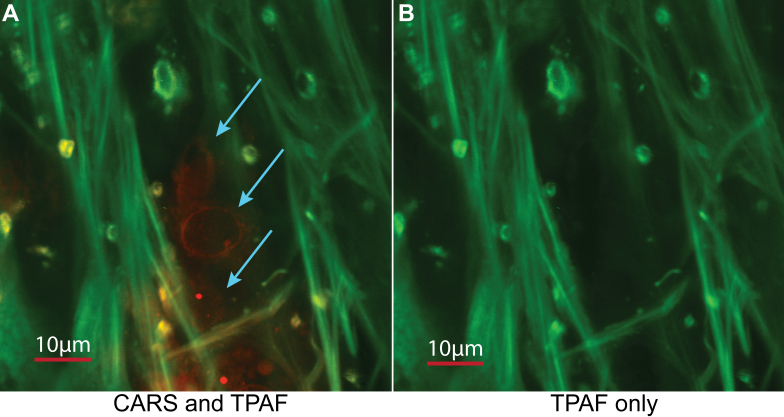
Label-free imaging of TM cells using CARS and collagen extracellular matrix using TPAF. The image is taken using a 60× 1.2 NA water objective with 3× digital zoom. (RED: CARS. Green: TPAF). **A**: Both CARS and TPAF channels are displayed in the image, clearly showing the TM cells in the CARS channel with arrows indicating the TM cells. **B**: Only the TPAF channel is displayed and TM cells are not observed, indicating that signal of the TM cells are unique to the CARS signal.

In Figure 4, the scanning magnification of the image has been increased 3 times using the 60× objective (180× final magnification) to show a region with several TM cells in close proximity. At this resolution, the outer cell membrane structure can be clearly observed with no additional intracellular membrane structure. Altogether, the data demonstrates the efficacy of CARS and its ability to selectively visualize the TM cells as the cell membrane structure is only displayed in the CARS channel while only the collagen fiber extracellular matrix structure is shown in the TPAF imaging channel.

## Discussion

Both CARS and TPAF are powerful nonlinear label-free optical imaging techniques that have been used to image periocular structures in the past [17]. In this report, we have illustrated the use of a unique approach of simultaneous CARS-TPAF modalities to produce images of the TM and surrounding tissues with excellent resolution. The CARS laser photon energy difference was set to the CH_2_ vibrational frequency, allowing the detection of the various lipid molecules that compose the plasma membrane of living cells. In addition, the excitation photons used in CARS microscopy can be simultaneously absorbed and autofluoresce by the collagen molecules through TPAF. Combining the two techniques, the collagen structures and the TM cells can be readily observed without exogenous labeling.

Inadequate drainage of aqueous humor is believed to lead to elevated IOP, a leading risk factor for the development of glaucoma. Visualizing individual TM endothelial cells may be an important clinical metric of disease progression due to the known decrease in TM cells that occurs in the setting of glaucoma [1,18]. Although imaging modalities exist that image Schlemm’s canal, there are no conventional ophthalmic techniques for imaging individual TM cells in vivo [19]. Imaging devices such as optical coherence tomography as well as ultrasound biomicroscopy lack subcellular resolution and/or tissue penetrating capabilities to produce data useful for the evaluation of TM cell function.

We used TPAF and CARS techniques to image deeply into the native TM region of the human eye. Images were taken at multiple depths, allowing for visualization of the tissue in three dimensions. Similar images can be achieved with histological sections or EM ultra-thin sections; however the method described here has the advantage of being performed on unprocessed, unfixed tissue. Using TPAF and CARS for imaging the TM results in images free from the potential distortions that result from infusion of fixatives and treatment with alcohols. We anticipate this new label-free imaging technique can be used to help elucidate mechanisms of aqueous outflow through the conventional outflow system of the eye and to quantify the effects of TM cell number and distribution on the glaucomatous disease process. In vivo studies are now underway in mice to refine our technique and reports will be forthcoming.
